# A Generative Model to Synthesize EEG Data for Epileptic Seizure Prediction

**DOI:** 10.1109/TNSRE.2021.3125023

**Published:** 2021-11-02

**Authors:** Khansa Rasheed, Junaid Qadir, Terence J. O’Brien, Levin Kuhlmann, Adeel Razi

**Affiliations:** 1 Department of Electrical EngineeringInformation Technology University (ITU) 243044 Punjab Lahore 54000 Pakistan; 2 Department of Computer Science and EngineeringCollege of Engineering 90938 Qatar University Doha Qatar; 3 Department of NeuroscienceCentral Clinical SchoolMonash University 2541 Melbourne VIC 3800 Australia; 4 Faculty of Information TechnologyMonash University 2541 Clayton VIC 3800 Australia; 5 Turner Institute for Brain and Mental Health and Monash Biomedical Imaging, Monash University 2541 Clayton VIC 3800 Australia; 6 Wellcome Centre for Human NeuroimagingUCL London WC1E 6BT U.K; 7 CIFAR Azrieli Global Scholars ProgramCIFAR 145760 TorontoONM5G 1M1Canada

**Keywords:** Epileptic seizure, EEG, machine learning, deep learning, transfer learning, adversarial networks

## Abstract

Objective: Scarcity of good quality electroencephalography (EEG) data is one of the roadblocks for accurate seizure prediction. This work proposes a deep convolutional generative adversarial network (DCGAN) to generate synthetic EEG data. Another objective of our study is to use transfer-learning (TL) for evaluating the performance of four well-known deep-learning (DL) models to predict epileptic seizure. Methods: We proposed an algorithm that generate synthetic data using DCGAN trained on real EEG data in a patient-specific manner. We validate quality of generated data using one-class SVM and a new proposal namely convolutional epileptic seizure predictor (CESP). We evaluate performance of VGG16, VGG19, ResNet50, and Inceptionv3 trained on augmented data using TL with average time of 10 min between true prediction and seizure onset samples. Results: The CESP model achieves sensitivity of 78.11% and 88.21%, and false prediction rate of 0.27/h and 0.14/h for training on synthesized and testing on real Epilepsyecosystem and CHB-MIT datasets, respectively. Using TL and augmented data, Inceptionv3 achieved highest accuracy with sensitivity of 90.03% and 0.03 FPR/h. With the proposed data augmentation method prediction results of CESP model and Inceptionv3 increased by 4-5% as compared to state-of-the-art augmentation techniques. Conclusion: The performance of CESP shows that synthetic data acquired association between features and labels very well and by using the augmented data CESP predicted better than chance level for both datasets. Significance: The proposed DCGAN can be used to generate synthetic data to increase the prediction performance and to overcome good quality data scarcity issue.

## I. Introduction

A sudden abnormal, self sustaining electrical discharge in the cerebral networks of the brain is a cause of epileptic seizures (ES). The attack may occur at any time on any day. The unpredictability of duration, seriousness, and time of attack makes it very difficult for patients to perform everyday chores and on occasions can be life-threatening. According to the World Health Organization (WHO), 70 million people around the globe suffer from epilepsy with around one-third of these patients resistant to anti-epileptic medication [1]. The early prediction of these attacks before they occur will be helpful for the patients to take precautionary measures and potentially allow the implementation of preventative therapies.

Electroencephalography (EEG) is used for measuring and monitoring brain activity before, during, and after ES and is widely used to predict seizures. Machine Learning (ML) based prediction algorithm uses the hand-crafted features of EEG from the time-domain, frequency-domain, or time-frequency domain to make predictions. Previously, researchers have evaluated various features—such as Kolmogorov entropy [2], largest Lyapunov exponent (LLE) [3], phase synchronization of different EEG channels [4], and correlation density—to perform seizure prediction [5]. In 2014 and 2016, contests of (epileptic) seizure prediction were held by the American Epilepsy Society and Melbourne University. These competitions were open to the EEG feature (for seizure prediction) computing algorithm or ML models trained on the extracted features. However, the preferable performance of submitted algorithms was based on the extraction of various features and combinations of classifiers. The best combination of features and classifiers are still not known for each patient. These algorithms were also not generalizable and required significant changes for every new patient and new data in practical application [6]. Because of these shortcomings of feature engineering methods, more generalized methods for seizure prediction are required.

Deep learning (DL) algorithms are beneficial in the sense of automatic feature extraction from the data [7]. Over the past few years, researchers have applied several DL methods to predict epileptic seizures [8]–[10]. However, these DL algorithms require an extensive amount of labeled data to produce effective results. Researchers typically use scalp EEG, where signals are collected from the wearable sensors placed on the scalp, or intracranial EEG (iEEG), signals collected by placing the electrode on the exposed surface of the brain through surgery, for the ES prediction. iEEG data gives the high temporal resolution and frequency information of brain activity with a high signal-to-noise ratio as compared to scalp EEG. However, the acquisition of iEEG data is challenging due to surgery and implantation risks. Moreover, the characteristics of seizures may change over time, not only for each patient but also for an individual. To achieve good performance, the seizure dataset must contain all the possible characteristics for which we required long-term continuous recordings of EEG signals.

Despite decades of research on ES prediction, the field still lacks the availability of long-term continuous good quality patient EEG data. The collection of data for the first-in-man study of Cook *et al.* [11] and the EPILEPSIAE dataset [12] are steps towards solving this problem but the latter dataset is not free. The former dataset consists of almost one-year-long iEEG recordings of patients (the dataset of 3 most critical patients from this collection is available as Epilepsyecosystem dataset, which we used in our study). There are additional practical challenges related to data collection such as inducing headache, surgical infection, discomfort in neck and head due to implanted device, and accumulation of fluid around implantation area of the brain. Data collection procedures are very costly because medical experts are required to avoid the aforementioned medical situations.

**Fig. 1.**
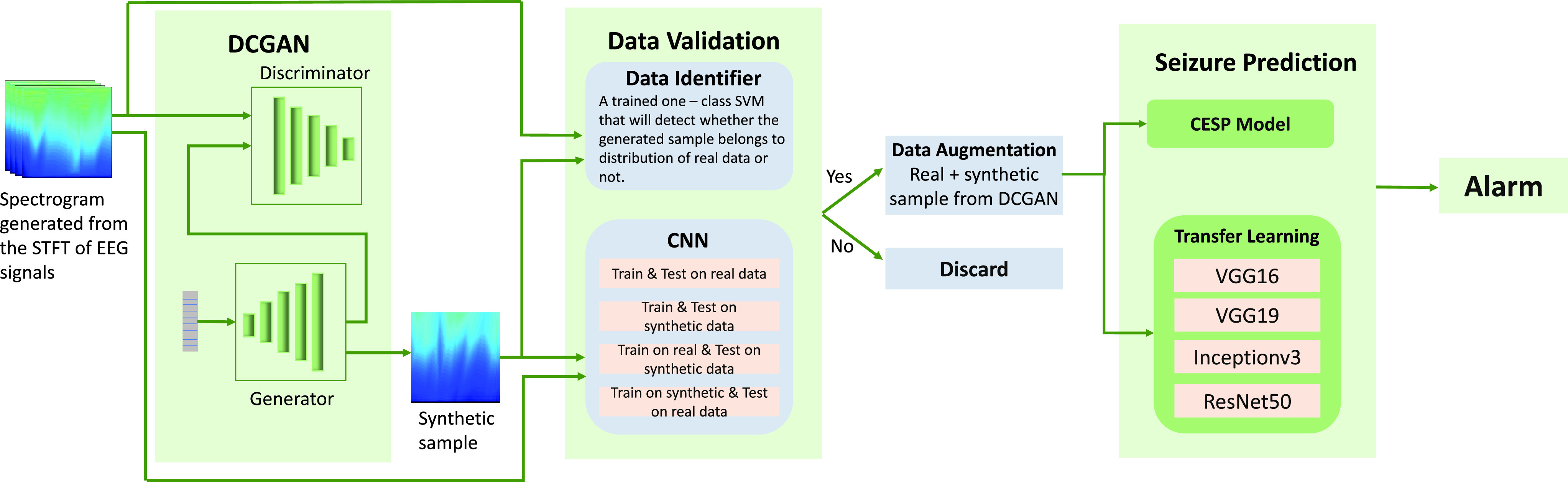
Pipeline of the methodology used in the paper.

Apart from these challenges related to the acquisition of long-term continuous EEG/iEEG signals, another challenge is the imbalanced nature of the dataset due to the low frequency of seizure occurrence with a preponderance of interictal (non-seizure) samples. The scarcity of good quality epilepsy data recordings arises due to these challenges and motivates our work. We propose the generation of artificial synthetic data as a solution to this problem. In this paper, we will examine that how a DL algorithm can be used as a generalized model for artificial EEG data generation? We will also verify how much effective the artificial EEG data is for seizure prediction? Our major contributions in this paper are as follows:

1) We propose a deep convolutional generative adversarial network (DCGAN) to resolve the EEG data scarcity problem. As a proof of principle, we generate synthetic scalp EEG and iEEG data of each patient by training the DCGAN model on the Epilepsyecosystem iEEG data and CHB-MIT scalp EEG data separately in a patient-specific way.
2) To evaluate the effectiveness of simulated data, we then use two methods: classical ML method of one-class SVM, and secondly a CNN classifier—which we refer to as convolutional epileptic seizure predictor (CESP)—for seizure prediction in the last block of Figure 1). We train CESP on the real data and test on synthesized data (iEEG data generated from DCGAN). The idea of augmenting the real data with generated data also improved the performance of CESP.
3) We also evaluate data augmentation with the well-known technique of transfer learning (TL). We trained the popular DL models ResNet50, Inceptionv3, VGG16, and VGG19 on the large amount of already generated synthesized data. After the training, we fine-tuned these pre-trained models on the real data to develop the patient-specific prediction algorithm.

To the best of our knowledge, this is the first study on the generation of synthetic scalp EEG and iEEG data and the use of TL with data augmentation performed by generative methods for seizure prediction. Figure 1 summarizes the method we have developed.

The remaining paper is organized as follows: Section II covers the review of previously used methods. Section III provides face validation, i.e., the detailed methodology we propose along with the used dataset. Section IV provides construct validation, which comprises the results obtained from the proposed methods and comparison of the results with previous works. The paper is concluded in Section VI.

## II. Related Work

EEG is a complex and challenging functional brain mapping modality to handle due to the presence of noise and various measurement and physiological artifacts. Pre-processing of EEG data for noise and artifact removal is an involved and time-consuming exercise that greatly compromises the utility of online development of EEG based ES prediction solutions [13]. Furthermore, the imbalance EEG data leads to overfitting and wrong predictions.

To overcome the problem of imbalanced dataset, Troung *et al.* generated pre-ictal samples by sliding a 30sec window over the pre-ictal samples along the time axis [8]. They trained a three-layered convolutional neural network (CNN) on the spectrograms generated from the short-time Fourier transform (STFT) of the Freiburg hospital iEEG database and the CHBMIT scalp EEG database. They achieved 89.8% sensitivity with 0.17 false prediction rate (FPR/h) for 5 min of Seizure Prediction Horizon (SPH). SPH is defined as the time interval between the alarm raised in the anticipation of impending seizure onset and the actual start of the ictal state [14]. To deal with the imbalance dataset, Khan *et al.* [9] down-sampled the inter-ictal class by randomly picking the samples from the data. They achieved 87.8% sensitivity and 0.142 FPR for 10 min SPH with the CHBMIT and MSSM databases. They transformed the EEG data into wavelet tensors before training the CNN classifier. Daoud and Bayoumi [15] also used this down-sampling method of inter-ictal samples to overcome the problem of imbalanced data.

Another work, in which Troung *et al.* [10] proposed the solution to the unavailability of unlabelled EEG data, they again used the overlapping sampling method to generate pre-ictal samples (they used this method in their previous work [8]). They trained a generative adversarial network (GAN) to perform unsupervised training on the spectrogram of STFT of EEG. Then, they used the features learned from the discriminator to predict seizures. They measured the area under the receiver operator characteristic (ROC) curve as a performance measure with 5 min SPH and seizure occurrence period (SOP), the time interval during which seizure occurrence is expected, of 30 min. They achieved 77.68% AUC for the CHBMIT scalp EEG data, 75.47% AUC on the Freiburg hospital data, and 65.05% AUC with the EPILEPSIAE database.

Data augmentation is a traditional solution to the intricacy of a small dataset. Zhang *et al.* addressed the problem of an imbalanced dataset by generating pre-ictal samples by randomly combining the segments of original pre-ictal samples and augmented the real data with the generated samples [16]. They extracted the time and frequency domain features using wavelet packet decomposition and common spatial patterns (CSP). Then they fed the extracted features to the shallow CNN classifier to predict the seizure. They achieved a sensitivity of 92.2% and 0.12 FPR/h on the 23 patients of the CHB-MIT dataset. Synthetic monitoring oversampling (SMOTE) is another technique used in literature as a solution to the imbalance dataset. This method generates the samples of minority class by focusing on the feature space of samples. Stojanović *et al.* used the SMOTE method to generate pre-ictal samples of EPILEPSIAE and Epilepsyecosystem datasets [17]. They achieved a sensitivity of 69% and 95% for the Epilepsyecosystem dataset and EPILEPSIAE dataset respectively. They achieved these results by extracting 12 non-negative matrix features and training support vector machines (SVM) on these features.

Random selection of data samples is not a recommended solution because one may lose the useful information present in the samples which is discarded during random selection. On the other hand, oversampling of pre-ictal data is a better solution compared to the imbalanced dataset. However, techniques used in literature, i.e., SOMTE, data-segmentation, and sliding window generate samples with no new information. The sliding window method is not useful if we have to generate a large number of samples because the overlapping windows may lead to redundant samples with repeated information. SMOTE generates the samples by translating, rotating, and adding noise to the original samples. So, the samples generated using SMOTE can be misleading due to the presence of noise. In contrast to these techniques, we used a DL-based method for the artificial generation of pre-ictal data. Using our method, we can generate samples similar to the original samples without any added noise. Our method eliminates the requirement of availability of original data to generate new samples once the model is trained. In this manner, we can generate as many samples as we want without the dependency on real data.

## III. Methodology

In this section, we describe the datasets used in the study, pre-processing of these datasets, and then we present the face validation of our proposed methodology.

### A. Dataset

We are using two datasets for this work: the CHB-MIT dataset [18] and the Epilepsyecosystem dataset [11] (summarized in Table I). We trained the DCGAN on both datasets separately to generate the samples of scalp EEG and iEEG signals. After the generation of synthetic data, we augmented the real data of both datasets with the synthetic samples. We then employed the idea of transfer learning (TL) on various DL models using augmented Epilepsyecosystem dataset. We also evaluated the performance of the CESP model on both augmented datasets.

**TABLE I:** Summary of the Datasets Used in the Paper

Dataset	EEG type	No. of patients	No. ofchannels	No. ofseizures	Duration average (hr)
CHB-MIT	scalp	13	22	64	1-4
Epilepsy–ecosystem	iEEG	3	16	1362	10608

The Epilepsyecosystem dataset recorded at St Vincent’s Hospital in Melbourne, Australia is used for the experiments. The dataset contains the intracranial EEG (iEEG) signals of three patients (all female). Data of each patient contains signals from 16 electrodes sampled at 400Hz sampling frequency. Data is segmented in 10 min long pre-ictal and interictal intervals. Pre-ictal intervals are selected from the one hour earlier recordings of every seizure with a five-minute seizure horizon while interictal intervals are segmented from randomly selected one-hour recording blocks at least four hours away from any seizure.

The CHB-MIT dataset consists of the 844h long continuous scalp EEG data of 23 patients with 163 episodes of seizure. Scalp EEG data were collected using 22 electrodes at a sampling rate of 256 Hz. We segmented the interictal periods that are at least 4 hours away before a seizure onset and after a seizure ends. We are interested in anticipating the leading seizures, therefore for two or more consecutive seizures that are less than 30 min apart from each other, we considered these as only one seizure. Moreover, we only considered patients with no more than 10 seizures per day for the ES prediction because it is not very crucial to predict ES for patients that have a seizure every 2 hours on average. With the preceding criteria, there are 13 patients with an adequate amount of data, i.e., have at least 3 leading seizures and 3h of the interictal period.

### B. Data Preparation

As we are using a CNN model for predicting a seizure, we need to convert the time-series data into a matrix (image-like format). We have applied the short-time Fourier transform (STFT) to transform EEG signals into spectrograms containing the time and frequency axes. We applied STFT on each electrode of the EEG signals of both datasets with a 1 min window length with no overlaps. Some patients of CHB-MIT data have less than 22 channels, i.e., Pat9 and Pat13 have 21 and 17 channels respectively. To keep our synthetic samples independent of such data specifications, spectrograms of all electrodes were concatenated vertically to obtain the final spectrogram of shape $(X \times Y \times 3)$, where X and Y are time and frequency dimensions. To get all samples of the same size, we resized all spectrograms by setting the values of X and Y equal to 256. EEG data was contaminated by the power line noise at 60Hz for the CHB-MIT dataset and 50Hz for the Epilepsyecosystem dataset. We can remove this by eliminating the frequency components in the range of 47–53 Hz and 97–103 Hz for 50 Hz power frequency and frequency components in the range of 57–63 Hz and 117–123 Hz for 60 Hz power frequency. We removed the line noise in both datasets using the Butterworth infinite impulse response.

### C. Synthetic Data Generation

We use a DCGAN to generate synthetic iEEG and scalp EEG data. Here we only describe it for the Epilepsyecosystem dataset. The same description is implemented for the CHB-MIT dataset. The Generator takes a 100 dimensional randomly generated samples from the standard Gaussian distribution of zero mean and standard deviation of one as an input. The input layer is a dense hidden layer. The output dimension of the first hidden layer is 4096 which is reshaped to $4 \times 4 \times 256$. The dense layer is succeeded by 6 de-convolutional layers with a stride size of $2 \times 2$, filter size $5 \times 5$, and the same padding. Number of filters in first de-convolutional layer are 256 and 128 in all other de-convolutional layers. The output of the generator is the same as the spectrograms generated by the STFT ($256 \times 256 \times 3$).

We configured the discriminator to distinguish the synthetic iEEG data from the real data. The discriminator consists of 4 convolutional layers with 256, 128, 64, and 32 number of filters. The filter size in the convolutional layers is 5 x 5, with a stride of $2\times 2$, and the same padding. While training, the task of the discriminator is to detect whether the spectrograms generated by the Generator are real or fake. The Generator updates its parameters to generate the spectrograms that are not distinctive from real spectrograms [19].

The equations of discriminator loss $D_{loss}$ and the Generator loss $G_{loss}$ are defined as [19]:

\begin{align*} D_{loss}=&\frac {1}{n}\sum _{j=1}^{n}\left [{ \log D(x^{(j)})+\log (1-D(G(z^{(j)}))) }\right]\quad \tag{1}\\ G_{loss}=&\frac {1}{n}\sum _{j=1}^{n}\log (D(x^{(j)}))\tag{2}\end{align*}

where $n$ is our batch size (32), $x$ is the real EEG spectrograms generated from the STFT, and $z$ is a random sample generator from the distribution $\mathcal {N}(0,1)$.

To overcome the problems of overfitting and convergence of discriminator, we configured an early-stopping function to have a check on $D_{loss}$ and $G_{loss}$. The early-stopping monitoring stops the training of DCGAN if, over subsequent $k$ training batches, the $D_{loss}$ keeps getting larger than the $G_{loss}$. We used a batch size of 32, $k=15$, Adam as an optimizer for gradient learning with 0.5 value of $\beta _{1}$, and $1e^{-3}$ learning rate. The value of $G_{loss}$ and $D_{loss}$ achieved the equilibrium point in around 3000 epochs with the early-stopping monitoring.

We trained the DCGAN on the Epilepsyecosystem iEEG data and the CHB-MIT scalp EEG data separately to obtain the synthetic iEEG and scalp EEG data. To achieve the best results, we trained the DCGAN with three different dataset settings: (i) training on all patients of the Epilepsyecosystem dataset; (ii) training on all patients of the CHB-MIT dataset; and (iii) training of DCGAN only on the pre-ictal class of data of all patients to generate the pre-ictal synthetic samples for data augmentation.

### D. One-Class SVM for Data Validation

Schölkopf *et al.* [20] proposed the idea of one-class SVM, which is an extension of a two-class SVM algorithm. One-class SVM is widely utilized to identify the outliers and anomalies in the dataset. A one-class SVM algorithm separates the data from the origin point by a wide margin in the higher dimensional feature space. Then the algorithm computes the surface of a hyperplane, which encloses the anomaly free data (+ve class). The data samples which are out of the hyperplane are outliers/anomalies. The radius of the hyperplane and the number of outliers/anomalies are hyperparameters to select through multiple experiments.

Let $X$ be the samples of the positive class of dataset such that $\left \{{ x_{i}\in R^{n}, i= 1\ldots l }\right \}$, the optimization equation of the algorithm is as follows:

\begin{align*}&\underset {\xi, \rho, z, b }{\text {minimize}}\quad \frac {1}{2} z^{T}z - \rho +\frac {1}{\nu l}\sum _{i=1}^{l}\xi _{i} \\&\text {subject to}\quad z^{T}\phi (x_{i})\geq \rho -\xi _{i}, \; \xi _{i}\geq 0.\tag{3}\end{align*}

In the optimization problem above, $\rho $ is the distance of hyperplane from the origin, $\xi _{i}$ are the hyperparameters, and $\nu \in (0,1]$ selects the fraction of outliers/anomalies outside the hyperplane. The decision function is:

\begin{equation*} \mathrm {sgn}\left({\sum _{i}^{l}\beta _{i}K(x_{i},x)-\rho }\right)\tag{4}\end{equation*}

Here $\beta _{i}$ are the Lagrange multipliers. We used the Radial Basis Function (RBF) as a kernel function in our experiments:

\begin{equation*} K(x_{i},x_{j}) = e^{-\gamma \left \|{ x_{i}-x_{j} }\right \|^{2}}\tag{5}\end{equation*}

In the above kernel function $\left \|{ x_{i}-x_{j} }\right \|^{2}$ is the Euclidean distance between two data points and $\gamma $ is a hyperparameter.

### E. CESP Model

**Fig. 2.**
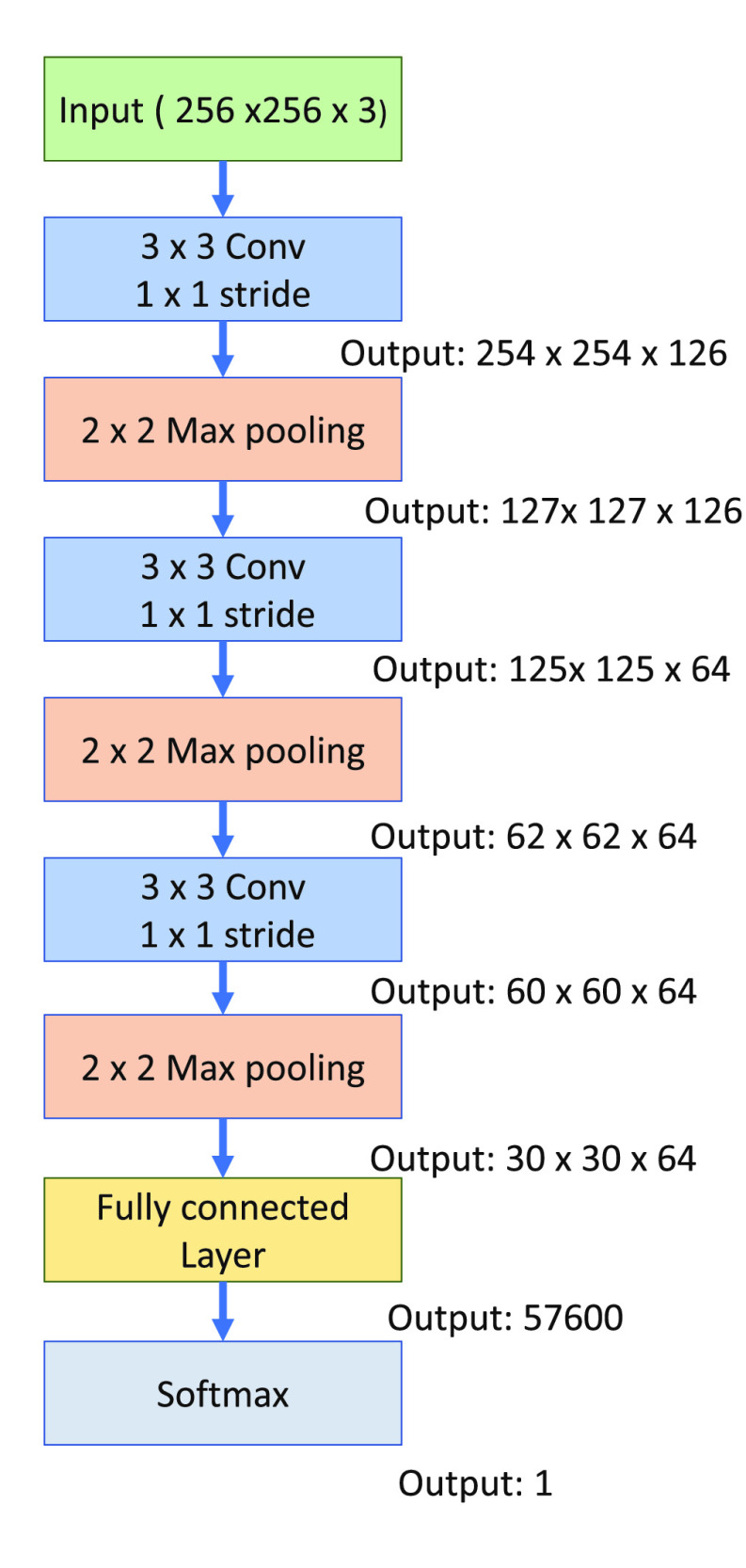
Architecture of convolutional epileptic seizure predictor (CESP).

CNNs models have been widely used for predicting the seizures successfully in literature [9], [21], due to their ability to learn local dependences of input and the fewer number of trainable parameters due to weight sharing. Based on the state-of-the-art performance of CNN in seizure prediction, we are using the CNN classifier here to evaluate the effectiveness of synthetic data generated from the above-mentioned architecture of DCGAN. The detailed architecture of the proposed model is described in Figure 2.

In this work, we used a CNN network that consists of 3 convolutional blocks followed by one fully connected (FC) layer. Each block contains a convolutional layer, a rectified linear unit (ReLu), and a max-pooling layer. The max-pooling technique enables the CNN model to learn temporal or spatially invariant features. The convolutional layers have the filter size of $3 \times 3$, stride $1 \times 1$, $2 \times 2$ size of max-pooling with 126, 64, and 64 number of filters respectively. The FC layers have a sigmoid activation function with output sizes of 32 and 2. We designed this particular architecture to achieve good performance with a simple model. We experimented with a different number of layers of the model and chose the described model of 3 convolutional layers providing good prediction results. To avoid the overfitting for the simple model, we evaluated the training process on the k-fold cross-validation. We used $k=10$ to split the training data into 90% for training and 10% for validation. We trained the CESP model for the binary-cross entropy loss on the Adam optimizer with a $1e^{-4}$ learning rate.

### F. Transfer Learning

With the rapidly growing applications of supervised learning in ML, a problem arises when we do not have a sufficient amount of labeled data for training. Transfer learning (TL) deals with this problem by leveraging the already available labeled data of relevant or similar tasks. Since 1993, TL has been used in discriminability-based transfer (DBT) algorithm [22], multi-tasking learning [23], cognitive science [24], detection of cancer subtypes [25], text classification [26], and spam filtration [27]. In another recent work, Bird *et al.* used TL between EEG signal classification and Electromyographic (EMG) signals [28].

We trained four well-known DL models: VGG16, VGG19, Inceptionv3, and ResNet50 on augmented EEG data. These are well-designed DL models intended to resolve problems of convolutional networks, i.e., vanishing gradient, degradation, long training time, and the large number of trainable parameters. We used the weights of these models trained on the ImageNet dataset as initial weights instead of training from random weights with learning rate of $1e^{-4}$. ImageNet dataset consists of more than 14 million images of almost 20,000 categories [29]. The dataset is one of the most widely used image repository and is freely available for training large neural network models.

### G. System Evaluation

Before evaluating the performance of the prediction algorithm, the SPH, and the Seizure Occurrence Period (SOP) need to be defined. For our work, we are using the definitions established in [14]. To make a correct prediction, a seizure must transpire after the SPH and within the SOP. A false alarm will be raised if the prediction algorithm gives a positive signal (seizure is going to occur) but there is no seizure during the SOP. For the best clinical use, the SPH must be long enough to give a patient sufficient time to take precautionary measures after the alarm is raised. We use sensitivity, FPR/h, specificity, and accuracy with SPH of 10 min and SOP of 30 min. For the Epilepsyecosystem the value of SPH is fixed only for the training dataset. As no information about the segmentation timing relative to seizures is provided for the test set, we cannot determine the exact value of SPH, we only have the information that we are 65 to 5 minutes away from the seizure.

We also compared the performance of CESP model trained on the augmented data with a random predictor. Using the method proposed by Schelter *et al.* [30], we computed the probability of alarm generation in the duration of SOP for a given value of FPR:

\begin{equation*} {P}\approx 1-e^{-FPR\times SOP}.\tag{6}\end{equation*}

Then the probability to predict at least ${n}$ out of ${N}$ independent seizure events at random can be calculated using the following equation:

\begin{equation*} {p}= \sum _{k\geq n}\binom {N}{k}{P}^{k}(1-{P})^{N-k}.\tag{7}\end{equation*}

Using the FPR value of each patient and the number of true predictions using CESP (${n}$), We computed the p-value for each patient. The null hypothesis is that our algorithm cannot detect a pre-ictal state with a performance above chance level. With the results discussed in next section (Sec IV) we show that for the significance level of $\alpha = 0.05$, our approach performed better than a chance level predictor.

### H. Implementation Details

We performed all our experiments on a core i3 processor with 16 GB RAM having Quadro M5000 8GB GPU card. It took almost two days to train our DCGAN model for 3000 iterations. Once the DCGAN is trained, we can generate a synthetic sample (spectrogram of 10 min long iEEG/EEG sample having size of $256 \times 256 \times 3$) in less than a second. Our CESP model took four hours for training on five times the augmented data of Epilepsyecosystem dataset and two hours for the CHB-MIT dataset. For predicting a seizure, our CESP model took only 0.4 msec.

## IV. Results

To generate the synthetic data, we trained the DCGAN on the iEEG data of Epilepsyecosystem and the scalp EEG data of CHB-MIT datasets. In this section, we test the effectiveness of synthetic data using the one-class SVM and CESP. The selection of samples of generated data is based on the results of one-class SVM and CESP model. These selected samples were then used for data augmentation for ES prediction. For the CESP we applied different combinations of real and synthetic data while testing and training. Detailed results are described in Table II, III.

**TABLE II:** Validation of Synthesized Pre-Ictal Samples of Scalp EEG Data Using Different Combinations of Synthesized and Real Data for Training and Testing on CESP. Synthesized Data Generated From the DCGAN trained on the CHB-MIT dataset. The Comparison of Results of i) Test & Train on Real Data (TRTR) and ii) Train on Synthetic Data & Test on Real Data (TSTR), Shows That the Synthetic Data Has Fully Captured the Correlation Between the Features of Data and the Labels. Legends: TRTR = Train & Test on Real Data, TSTR = Train on Synthesized Data & Test on Real Data, TRTS = Train on Real Data & Test on Synthesized Data, TSTS = Train & Test on Synthesized Data, Sen = Sensitivity, Spec = Specificity, Acc = Accuracy

Patients	TRTR				TSTR				TRTS				TSTS			
	Sen(%)	FPR/h	Spec (%)	Acc(%)	Sen (%)	FPR/h	Spec (%)	Acc(%)	Sen (%)	FPR/h	Spec (%})	Acc(%)	Sen (%)	FPR/h	Spec (%)	Acc(%)
Pat1	91.59	0.19	81	92.03	88.15	0.24	76	90.18	88.09	0.25	75	90.85	90.58	0.18	82	91.57
Pat2	75.2	0.01	99	89.24	70.59	0.04	96	85.49	68.13	0.01	99	84.08	71.23	0.02	98	88.93
Pat3	95.11	0.14	86	96.11	96.36	0.17	83	96	95.21	0.15	85	95.55	95	0.14	86	94.89
Pat5	89.1	0.15	85	88.36	78.59	0.19	81	83.19	88.64	0.18	82	86.52	90.26	0.16	84	89.91
Pat9	65.23	0.01	99	78.51	70.51	0.02	98	80.26	62	0.00	100	89.97	71.87	0.00	100	89.99
Pat10	88.56	0.16	84	89.91	90.23	0.15	85	89.99	85.27	0.13	87	88.92	92.58	0.12	88	90.56
Pat13	90.14	0.22	78	91.15	89.36	0.19	81	91.05	88.06	0.20	80	90.18	92.41	0.18	82	91.07
Pat14	99.01	0.15	85	96.34	98.24	0.16	84	95.74	100	0.12	88	98.43	100	0.11	89	97.87
Pat18	95.41	0.21	79	89.37	93.36	0.25	75	88.43	96.28	0.24	76	92.09	98.99	0.20	80	91.99
Pat19	90.38	0.29	71	92.31	91	0.30	70	91.50	91.45	0.31	69	90.83	93.89	0.25	75	91.38
Pat20	87.58	0.18	82	90.84	88.45	0.14	86	89.09	89.63	0.16	84	90.44	91.56	0.15	85	90.59
Pat21	93.57	0.00	100	94.83	93.79	0.02	98	92.58	92.03	0.00	100	95.14	96.32	0.01	99	96.46
Pat23	100	0.05	95	97.62	98.1	0.03	97	97.32	99.56	0.07	93	92.21	100	0.02	98	91.17
Average	89.28	0.13	87	91.24	88.21	0.139	86	90.03	88.02	0.14	86	91.17	90.9	0.11	89	92.02

**TABLE III:** Validation of Synthesized Pre-Ictal Samples of iEEG Data Using Different Combinations of Synthesized and Real Data for Training and Testing on CESP. Synthesized Data Generated From the DCGAN trained on Epilepsyecosystem Dataset. The Comparison of Results Of: i) Test & Train on Real Data (TRTR) and ii) Train on Synthetic Data & Test on Real Data (TSTR), Shows That the Synthetic Data Has Fully Captured the Correlation Between the Features of Data and the Labels

Data	Patient 1				Patient 2				Patient 3			
	Accuracy(%)	Sensitivity(%)	FPR/h	Specificity(%)	Accuracy(%)	Sensitivity(%)	FPR/h	Specificity(%)	Accuracy(%)	Sensitivity(%)	FPR/h	Specificity(%)
TRTR	72.59	78.01	0.4	60	73.00	82.16	0.31	69	69.81	75.00	0.22	78
TSTR	70.12	76.59	0.38	62	74.21	81.46	0.25	75	71.24	76.30	0.20	80
TRTS	68.98	75.30	0.39	61	71.18	79.23	0.28	72	70.00	72.39	0.23	77
TSTS	72.00	79.18	0.39	61	72.99	82.00	0.22	78	70.98	75.11	0.21	79

For the selection of correct generated samples, we trained the one-class SVM algorithm on the datasets of real EEG signals separately. We considered that the real data is a positive class and the anomalous data is a negative class. The algorithm learns the distribution of real EEG data and classifies the generated data sample in a positive class or negative class. After training one-class SVM on real data, we tested it for the synthesized samples. We selected those synthesized samples which belonged to the positive class and discarded the samples that belonged to the negative class (see Figure 1).

**Fig. 3.**
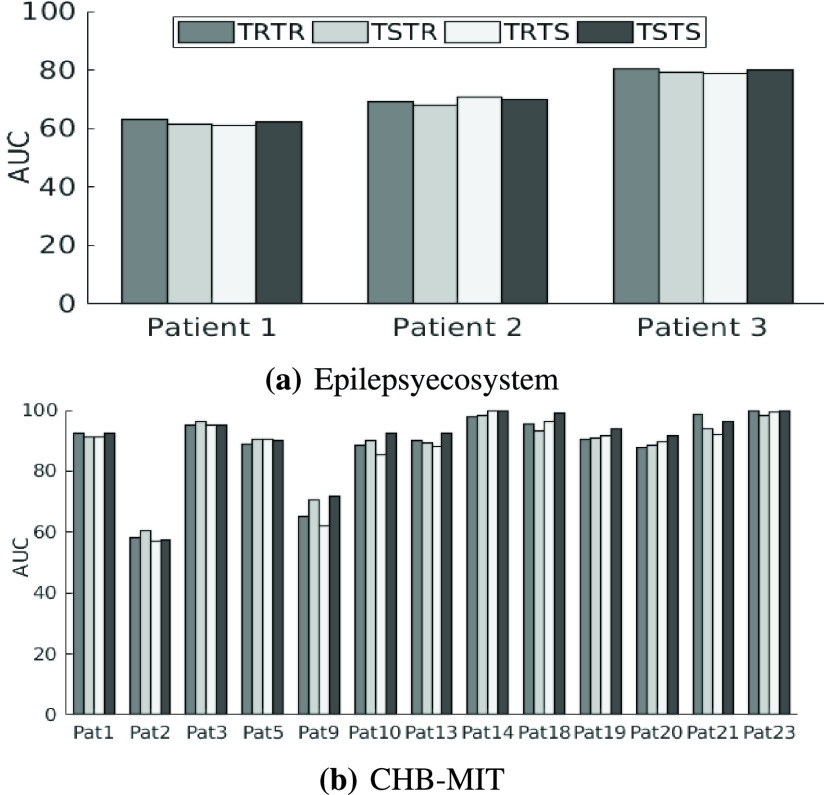
Seizure prediction performance (AUC) of CESP using different combinations of real (iEEG data of Epilepsyecosystem, scalp EEG data of CHB-MIT) and synthetic (generated iEEG data from DCGAN trained on Epilepsyecosystem dataset, generated scalp EEG data from DCGAN trained on the CHB-MIT dataset) for testing and training. **Legends**: **TRTR** = train & test on real data, **TSTR** = train on synthesized data & test on real data, **TRTS** = train on real data & test on synthesized data, **TSTS** = train & test on synthesized data.

To further validate the selected samples, we performed testing and training of the CESP model for four data combinations. We train and test the model on real data (TRTR) to check and compare the performance of the model with generated data. Then, we test the model trained on real data for samples of synthetic data (TRTS). We also trained the model on synthetic data and evaluated the performance on the real data (TSTR) to validate the selected samples of synthetic data. For all experiments, we selected test samples from the period that is not used in training, i.e., the training and testing samples are from totally different time sections. We selected 25% of the data as test data for all the evaluations and experiments. These samples are randomly selected from any time period while ensuring that the time period is not used in training samples. The results of these experiments for the Epilepsyecosystem and CHB-MIT datasets are provided in Table II and III respectively. Figure 3 depicts the AUC of ES prediction results for multiple scenarios of testing and training. For the Epilepsyecosystem data, we achieved an average sensitivity of 78.39% for TRTR and 77.56% for TSTR. It shows that the generated samples selected from the one-class SVM are correct. Similar is the case for the CHB-MIT dataset, we achieved an average of 89.02% sensitivity for TRTR and 88.21% sensitivity for TSTR.

**Fig. 4.**
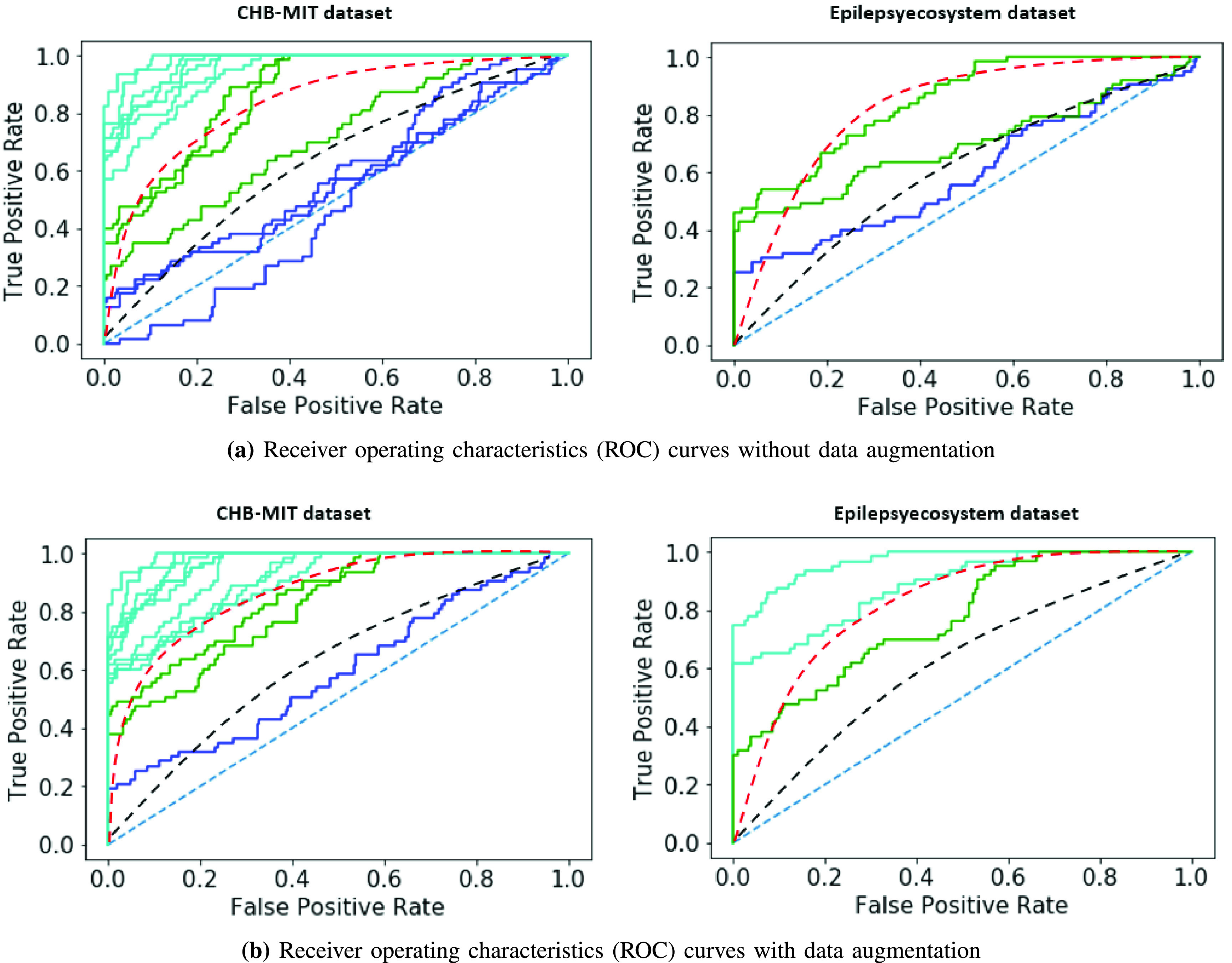
Receiver operating characteristics (ROC) curves of ES prediction performance testing for two datasets with and without augmentation: (a) without augmenting the datasets (b) with data augmentation using the synthetic samples from DCGAN. In these curves, each line represents a patient. Above the black dash line: good prediction performance; above the red dash line; very good prediction performance (adapted from [31]).

We experimented with the training of the CESP model on the $5\times $ and $3\times $ augmented Epilepsyecosystem and the CHB-MIT dataset respectively. Compared to the results achieved by using unaugmented data, data augmentation using DCGAN increased the sensitivity ~15% and AUC ~10% for Epilepsyecosystem dataset. For the CHB-MIT dataset, AUC increased ~6% for augmented data. Figure 4 demonstrates the overall ES prediction performance for two datasets with and without augmentation. Table IV shows the statistical comparison of CESP model trained on the augmented data using the proposed approach of data generation with the chance level predictor. Results indicate that the performance of CESP model for both datasets is better than the chance level predictor. We also compared the results of our data augmentation approach with the previous works in Table V. The comparison shows a significant increase in the prediction results by augmenting the data with synthetic samples for both datasets.

**TABLE IV:** For Epilepsyecosystem Dataset

(a) For CHB-MIT dataset	
Patient	${p}$-value
Pat1	<.001
Pat2	.029
Pat3	<.001
Pat5	<.001
Pat9	<.001
Pat10	.001
Pat13	<.001
Pat14	.002
Pat18	<.001
Pat19	0.03
Pat20	<.001
Pat21	<.001
Pat23	<.001

(b) For Epilepsyecosystem dataset.	
Patient	p-value
Patient1	.008
Patient2	<.001
Patient3	.0035

**TABLE V:** Comparison of Our Work With Previous Works

Year	Authors	No. of seizurein original dataset	Features	Classifier	Sensitivity(%)	FPR/h	Augmentation technique
CHB-MIT							
2017	Truong et al. [8]	64	STFT	CNN	81.2	0.16	Windowing
2020	Zhang et al. [16]	156	Common spatialfeatures	CNN	92	0.12	Division of a data sample into 3smaller segments and generation of artificial sample from random concatenation of these segments
2020	This work	64	STFT	CESP	96	0.05	Samples generated form DCGAN
Epilepsyecosystem							
2020	Stojanovic et al. [17]	692	Non-negativematrix factorization	SVM	69	0.76	Synthetic monitory over-sampling
2020	Ramy et al. [32]	1326	Continuous wavelettransform	Semi-DilatedCNN	89.52	N/M	Division of data into smaller segments
2020	This work	1326	STFT	CESP	92.87	0.15	Samples generated form DCGAN

**Fig. 5.**
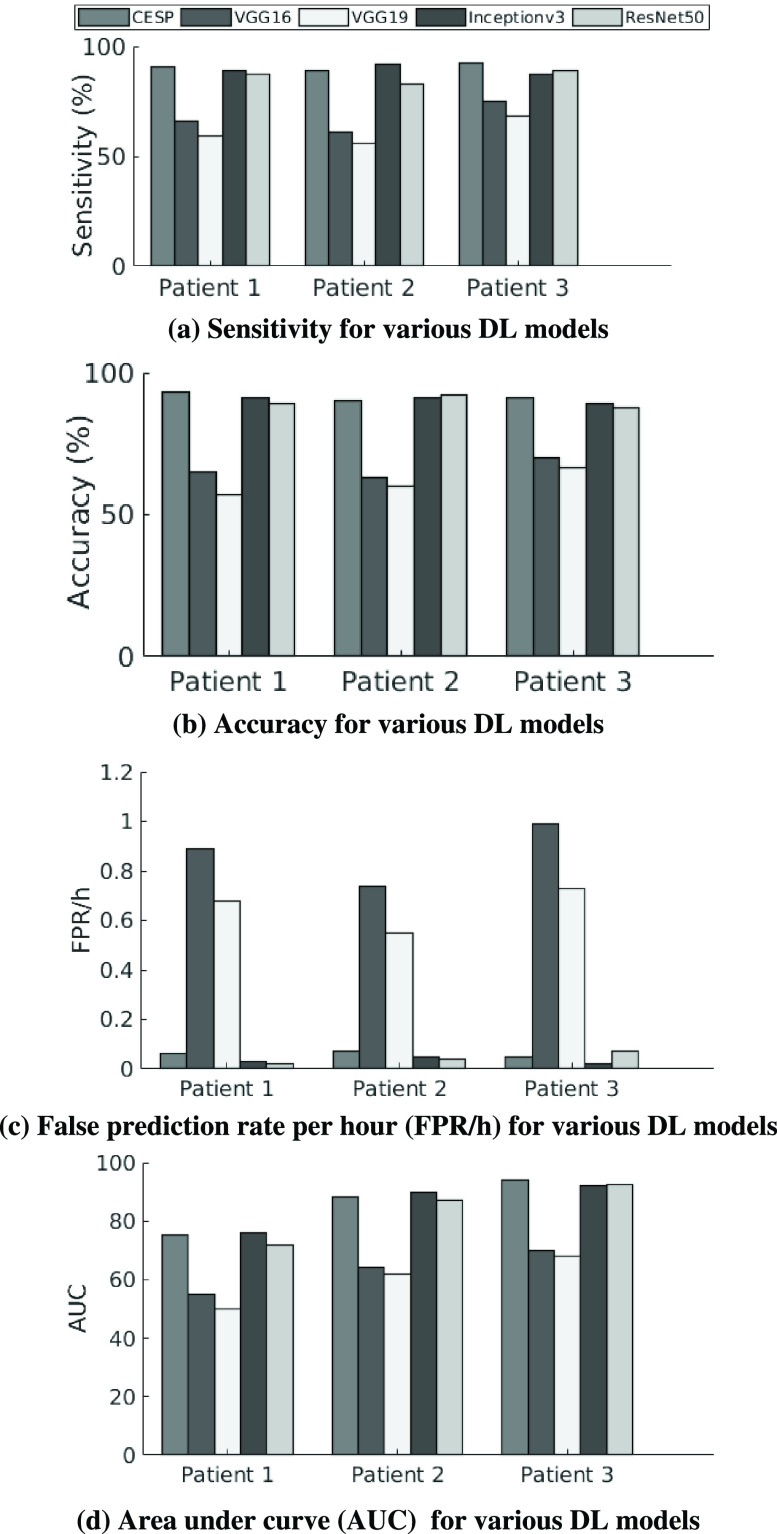
Comparison of performance of DL models trained on augmented **Epilepsyecosystem dataset** using transfer learning.

With the availability of augmented data, we evaluated the performance of four widely used DL models: VGG16, VGG19, Inceptionv3, and ResNet50. These models are used for image classification and weights of these models trained on the ImageNet dataset are available in Keras. We twice trained these models on augmented iEEG data with the pre-trained weights as initial weights. First we trained all models on the augmented data of all patients and then we fine-tuned the models in a patient-specific manner. Figure 5 depicts the results of seizure prediction with the models trained on Epilepsyecosystem augmented data. The performance of Inceptionv3 and ResNet50 is considerable as compared to the VGG16 and VGG19. VGG16 and VGG19 have more trainable parameters and required more training time. VGG16 and VGG19 overfit to the training data after some time of training which leads to the poor testing performance. However, the performance of other two models is good enough to use and explore the idea of training these models on adequate amount of data for seizure prediction in future.

Table VI shows the comparison of AUCs of TL models with the CESP. ${p}$-values for the VGG16, VGG19, and ResNet50 indicate a significant difference between the performance and AUC curves of prediction models. The performance of Inceptionv3 is best among the TL algorithms and approximately equal to the performance of the CESP model. However, the advantage of the CESP model is the low computational cost and complexity.

**TABLE VI:** Statistical Comparison (${p}$-Values) of Prediction Models. ${p}$-Values are Derived From the Single-TailedHanley-McNeil Test for Comparing AUCs

Patients	CESPVSVGG16	CESPVSVGG19	CESPVSInceptionv3	CESPVSResNet50
Patient1	<.000001^*^	<.000001^*^	.046	.000021^*^
Patient2	<.000001^*^	<.000001^*^	.085	.005^*^
Patient3	<.000001^*^	<.000001^*^	.059	.0025^*^

^*^ indicates significant p-values after penalizing for correction for multiple comparisons using Bonferroni for each patient at the level $\alpha=0.05 / 4$.

## V. Discussion

This work aimed to address the scarcity problem of good quality EEG data for ES prediction. With the advancement of DL techniques, high-quality artificial data generation is now possible. Deep generative models trained in an adversarial manner can simulate complex data distributions. In this paper, we presented a DL based generated model (DCGAN) that can generate the artificial EEG samples of patients. After measuring the quality of data using a traditional one-class SVM model and four different tests and training experiments, we augmented the real data with synthetic pre-ictal samples. We then trained CESP model on the augmented data and compared the performance of the model for ES prediction with previous works that used traditional augmentation techniques, i.e., SMOTE, moving windows, and data sampling. The comparison shows that the prediction performance using synthetic pre-ictal samples increased for both datasets. Figure 4 demonstrated that the ES prediction performance for all patients of both datasets increased than a chance level predictor by using the augmented data.

In contrary to previously used augmentation techniques, our technique generates artificial samples of data, which is also a solution to medical data sharing problems. Besides data acquisition difficulties, medical data sharing comes with the privacy-preserving issues. Researchers and hospitals cannot use the data without the permission of patients and ethical approval [33]. The synthetic data is not only used to augment the real data for performance improvement but also can be shared with researchers without privacy issues.

Previous works [17], [32] using SMOTE and data division into smaller segments techniques for augmentation achieved the sensitivity of 69% and 89.52% for the Epilepsyecosystem dataset. Authors in [17] achieved the results by extracting 12 non-negative matrix features and used 692 original pre-ictal samples while we trained DCGAN on the 1362 original samples without any feature extraction. The generalization of the prediction technique is a major pitfall for the majority of previous works. Moreover, these studies are based only on a single dataset which may raise the problem of generalized algorithms for practical use. The generalization of the model requires the prediction performance using different datasets. To overcome this problem, we validated our results on two datasets which shows the robustness of our proposal for generalized results. The work in [16] divided the original pre-ictal samples into 3 smaller samples and concatenated these samples with random selection to generate new data samples that belonged to the distribution of real data. Their augmentation technique provided significant results. However, they tested the augmentation method for only one dataset which had a small number of seizures per patient. They also employed feature engineering techniques on the data before feeding the data to CNN. Our data generation is more generalized and applicable to both iEEG and scalp EEG data.

In our proposed technique, as we are training DCGAN on the pre-processed clean data, our generated samples are not contaminated with artifacts and noise. So, the generation of clean samples in a more controlled environment is possible with the help of our proposed technique as compared to the previous augmentation methods. In this manner, our proposed method provides robustness against the noise present in the real data. However, if we compromise the cleaning and pre-processing of real data, it will affect the quality of generated samples and robustness to noise. The quality of generated samples and the robustness of our proposed technique also depend on the training iteration of DCGAN. In the capacity of our computational resources, we gained presentable results with 3000 iterations.

To date, researchers and developers are applying traditional ML and DL techniques for ES prediction. However, with the availability of computational resources and an ample amount of data, TL is an emerging technique to implement for problem-solving. In this paper, we presented the use of famous DL models, i.e., VGG16, VGG19, Inceptionv3, and ResNet50, for the first time to predict seizure. With the availability of augmented data, the experiments performed on these models showed significant results. More precisely, the performance results of Inceptionv3 and ResNet50 were accurate enough to use these pre-trained models for future works, e.g., use the model for new patients with fine tunning, use the model for prediction of other diseases with EEG signals, or use these pre-trained models for extracting significant features of EEG data and make predictions based on extracted features. Besides the promising results of Inceptionv3 and ResNet50, due to the computational complexities of these models, a clinically implantable device will not be an appropriate idea for these models. CESP model has less computational cost as compared to these two models. So, another future direction of TL work can be the reduction of complexities of these models while preserving the performance efficiency, i.e., we can utilize the features extracted from selected layers of models and predict seizures based on these features.

For the purpose of simplicity and ease of comparison, the research community stated the ES prediction problem as a binary classification problem. We have performed all the experiments with this binary classification assumption. However, for the clinical application of these experiments, the formulation of the problem is complex because seizures depend greatly on the type of seizure and patient characteristics such as the patient’s age and gender and the medication that the patient was taking during data acquisition. Moreover, we have not taken into consideration the impact of seizure type, characteristics of patients (age and gender), circadian profile of patients on the performance of GAN. For a more robust evaluation, of the prediction models, we need continuous seizure data because the pitfall of AUC performance metric is that it is typically calculated for the balanced dataset (same number of pre-ictal and inter-ictal samples). However, the actual data contained more inter-ictal periods as compared to pre-ictal events.

Unavailability of annotated data, privacy-preserving issues, and the ethical problems regarding private data sharing come with the promising results of ML and DL models. Artificial data generation is one solution to these problems. However, the time-series, i.e., EEG data for seizure prediction, contains information that appears many hours ago from the seizure event but is as useful as the information that appears one minute before the seizure. That is why the generation of continuous data is one of the future extension of our work. With the significant results of synthesized data samples for seizure prediction, the generation of artificial patient’s data is also possible. In this way, researchers can work with an ample amount of data of various patients to address the generalization problem.

## VI. Conclusion

The main aim of ES predictions research is to provide an accurate seizure warning system to patients to take precautionary measures ahead of seizure onset. However, such a solution is not yet available due to scarcity of suitable amount of seizure EEG data. In this paper, we proposed a deep convolutional generative adversarial network (DCGAN) model to overcome the hurdle of the unavailability of an extensive amount of EEG data. The proposed DCGAN model showed good generalization for the generation of both iEEG and scalp EEG data. Moreover, a convolutional epileptic seizure predictor (CESP), was proposed to validate the synthetic data, is also generalized to work with both types of EEG data. To measure the quality of synthetic data, we employed one-class SVM and training and testing of the CESP model with four combinations of real and synthetic data. The CESP model produced sensitivity of 78.11%, 88.21% and FPR/h of 0.27, 0.14 for training on synthesized and testing on real Epilepsyecosystem and CHB-MIT datasets respectively. These results are higher than the training and testing of the CESP model on real data. This shows that the synthetic samples fully captured the relationship between the features of data and the labels of pre-ictal samples. We also evaluated the performance of CESP, VGG16, VGG19, Inceptionv3, and ResNet50 on the augmented dataset using the concept of transfer learning (TL). Using the TL on augmented data, we showed that the Inceptionv3 performed very well with highest accuracy of 90.03% and 89.50% sensitivity. With these significant results, using TL, we can further explore this novel idea of employing TL techniques for ES prediction in the future work.

## References

[1] Promoting Mental Health: Concepts, Emerging Evidence, Practice: A Report of the World Health Organization, Department of Mental Health and Substance Abuse in Collaboration With the Victorian Health Promotion Foundation and the University of Melbourne, World Health Org., Geneva, Switzerland, 2005.

[2] K. Natarajan, R. Acharya, F. Alias, T. Tiboleng, and S. K. Puthusserypady, “Nonlinear analysis of EEG signals at different mental states,” Biomed. Eng. Online, vol. 3, no. 1, pp. 1–11, 2004. 15023233 10.1186/1475-925X-3-7 PMC400247

[3] J. Gotman and M. G. Marciani, “Electroencephalographic spiking activity, drug levels, and seizure occurence in epileptic patients,” Ann. Neurol., vol. 17, no. 6, pp. 597–603, Jun. 1985. 3927818 10.1002/ana.410170612

[4] L. D. Iasemidis, J. C. Sackellares, H. P. Zaveri, and W. J. Williams, “Phase space topography and the Lyapunov exponent of electrocorticograms in partial seizures,” Brain Topogr., vol. 2, no. 3, pp. 187–201, 1990. 2116818 10.1007/BF01140588

[5] M. Le Van Quyen, J. Martinerie, M. Baulac, and F. Varela, “Anticipating epileptic seizures in real time by a non-linear analysis of similarity between EEG recordings,” Neuroreport, vol. 10, no. 10, pp. 2149–2155, Jul. 1999. 10424690 10.1097/00001756-199907130-00028

[6] L. Kuhlmann, “Epilepsyecosystem.org: Crowd-sourcing reproducible seizure prediction with long-term human intracranial EEG,” Brain, vol. 141, no. 9, pp. 2619–2630, 2018. 30101347 10.1093/brain/awy210 PMC6136083

[7] K. Rasheed, “Machine learning for predicting epileptic seizures using EEG signals: A review,” IEEE Rev. Biomed. Eng., vol. 14, pp. 139–155, 2020. 10.1109/RBME.2020.3008792 32746369

[8] N. D. Truong, A. D. Nguyen, L. Kuhlmann, M. R. Bonyadi, J. Yang, and O. Kavehei, “A generalised seizure prediction with convolutional neural networks for intracranial and scalp electroencephalogram data analysis,” 2017, arXiv:1707.01976. 10.1016/j.neunet.2018.04.018 29793128

[9] H. Khan, L. Marcuse, M. Fields, K. Swann, and B. Yener, “Focal onset seizure prediction using convolutional networks,” IEEE Trans. Biomed. Eng., vol. 65, no. 9, pp. 2109–2118, Sep. 2017. 29989952 10.1109/TBME.2017.2785401

[10] N. D. Truong, L. Kuhlmann, M. R. Bonyadi, D. Querlioz, and O. Kavehei, “Epileptic seizure forecasting with generative adversarial networks,” IEEE Access, vol. 7, pp. 143999–144009, 2019.

[11] M. J. Cook, “Prediction of seizure likelihood with a long-term, implanted seizure advisory system in patients with drug-resistant epilepsy: A first-in-man study,” Lancet Neurol., vol. 12, no. 6, pp. 563–571, 2013. 23642342 10.1016/S1474-4422(13)70075-9

[12] M. Ihle, “Epilepsiae—A European epilepsy database,” Comput. Methods Programs Biomed., vol. 106, no. 3, pp. 127–138, 2012. 20863589 10.1016/j.cmpb.2010.08.011

[13] U. R. Acharya, S. V. Sree, G. Swapna, R. J. Martis, and J. S. Suri, “Automated EEG analysis of epilepsy: A review,” Knowl.-Based Syst., vol. 45, pp. 147–165, Jun. 2013.

[14] T. Maiwald, M. Winterhalder, R. Aschenbrenner-Scheibe, H. U. Voss, A. Schulze-Bonhage, and J. Timmer, “Comparison of three nonlinear seizure prediction methods by means of the seizure prediction characteristic,” Phys. D, Nonlinear Phenomena, vol. 194, nos. 3–4, pp. 357–368, 2004.

[15] H. Daoud and M. A. Bayoumi, “Efficient epileptic seizure prediction based on deep learning,” IEEE Trans. Biomed. Circuits Syst., vol. 13, no. 5, pp. 804–813, Oct. 2019. 31331897 10.1109/TBCAS.2019.2929053

[16] Y. Zhang, Y. Guo, P. Yang, W. Chen, and B. Lo, “Epilepsy seizure prediction on EEG using common spatial pattern and convolutional neural network,” IEEE J. Biomed. Health Informat., vol. 24, no. 2, pp. 465–474, Feb. 2020. 10.1109/JBHI.2019.2933046 31395568

[17] O. Stojanović, L. Kuhlmann, and G. Pipa, “Predicting epileptic seizures using nonnegative matrix factorization,” PLoS ONE, vol. 15, no. 2, 2020, Art. no. e0228025. 10.1371/journal.pone.0228025 PMC7001919 32023272

[18] A. H. Shoeb, “Application of machine learning to epileptic seizure onset detection and treatment,” Ph.D. dissertation, Massachusetts Inst. Technol., Cambridge, MA, USA, 2009.

[19] I. Goodfellow, “Generative adversarial nets,” in Proc. Adv. Neural Inf. Process. Syst., 2014, pp. 2672–2680.

[20] B. Schölkopf, R. C. Williamson, A. J. Smola, J. Shawe-Taylor, and J. C. Platt, “Support vector method for novelty detection,” in Proc. Adv. Neural Inf. Process. Syst., 2000, pp. 582–588.

[21] R. Hussein, M. O. Ahmed, R. Ward, Z. J. Wang, L. Kuhlmann, and Y. Guo, “Human intracranial EEG quantitative analysis and automatic feature learning for epileptic seizure prediction,” 2019, arXiv:1904.03603.

[22] L. Y. Pratt, “Discriminability-based transfer between neural networks,” in Proc. Adv. Neural Inf. Process. Syst., 1993, pp. 204–211.

[23] R. Caruana, “Multitask learning,” Mach. Learn., vol. 28, no. 1, pp. 41–75, 1997.

[24] L. Pratt, “Reuse of neural networks through transfer,” Connection Sci., vol. 8, no. 2, pp. 163–184, 1996.

[25] E. Hajiramezanali, S. Z. Dadaneh, A. Karbalayghareh, M. Zhou, and X. Qian, “Bayesian multi-domain learning for cancer subtype discovery from next-generation sequencing count data,” in Proc. Adv. Neural Inf. Process. Syst., 2018, pp. 9115–9124.

[26] C. B. Do and A. Y. Ng, “Transfer learning for text classification,” in Proc. Adv. Neural Inf. Process. Syst., 2006, pp. 299–306.

[27] S. Bickel, “ECML-PKDD discovery challenge 2006 overview,” in Proc. ECML-PKDD Discovery Challenge Workshop, 2006, pp. 1–9.

[28] J. J. Bird, J. Kobylarz, D. R. Faria, A. Ekárt, and E. P. Ribeiro, “Cross-domain MLP and CNN transfer learning for biological signal processing: EEG and EMG,” IEEE Access, vol. 8, pp. 54789–54801, 2020.

[29] J. Deng, W. Dong, R. Socher, L.-J. Li, K. Li, and L. Fei-Fei, “ImageNet: A large-scale hierarchical image database,” in Proc. IEEE Conf. Comput. Vis. Pattern Recognit., Jun. 2009, pp. 248–255.

[30] B. Schelter, “Testing statistical significance of multivariate time series analysis techniques for epileptic seizure prediction,” Chaos, Interdiscipl. J. Nonlinear Sci., vol. 16, no. 1, Mar. 2006, Art. no. 013108. 10.1063/1.2137623 16599739

[31] L. Kuhlmann, K. Lehnertz, M. P. Richardson, B. Schelter, and H. P. Zaveri, “Seizure prediction—Ready for a new era,” Nature Rev. Neurol., vol. 14, no. 10, pp. 618–630, Oct. 2018. 30131521 10.1038/s41582-018-0055-2

[32] R. Hussein, S. Lee, R. Ward, and M. J. McKeown, “Epileptic seizure prediction: A semi-dilated convolutional neural network architecture,” 2020, arXiv:2007.11716. 10.1016/j.neunet.2021.03.008 33780727

[33] W. G. van Panhuis, “A systematic review of barriers to data sharing in public health,” BMC Public Health, vol. 14, no. 1, pp. 1–9, Dec. 2014. 25377061 10.1186/1471-2458-14-1144 PMC4239377

